# A UK‐wide survey evaluation of capnography variation

**DOI:** 10.1111/anae.16603

**Published:** 2025-03-17

**Authors:** Andrew A. Shepherd, Jennifer L. Proc, Patrick A. Ward, Alistair F. McNarry, Mathew Lyons, the CaVa UK Collaborators, Thomas Colville, Ashoke J. Shah, Sarah Thornley, Ashley Davies, Sara Jones, Radharetnasivan Meiarasu, Ashton D. Dsouza, Simon N. Smith, Jay P. Dorman, Katy Foreman, Kirsty S. McClelland, Emily F. Reid, Ellen A. Gorman, Charlotte Spiers, Alison K. Hare, Thomas Abberton, Aisha Abdelrahman, Georgia Ashley, Michael Gardiner, Fiona Glarvey, Syed Hussain, Alexander Poyner, Rebecca Williams, Maeve Graham, Sandor Bako, Anish Chandrasekar, Laura Scott, Amy Spicer, Dan Turner, Sean Williams, Victoria Courtice, Kumar Saurabh, Vivek Trivedi, Tanveer A Ansari, Lucy Brown, Vaishali Vyas, Chloe Butel, Oskar Mahony, Amy M Nixon, Nathan P. Griffiths, Anand D. Padmakumar, Rhianna S. Jones, Jothika Thimappa, Lucy Rothwell, Mary Leese, Harriet L. White, Jessica H. Phillips, Mostafa A. Elsayed, John E. C. Melville, Tony Hodgetts, Angela E. Munteanu, Robert Brown, Aidan Butler, Angus Isham, Kate Lloyd, James G. Cowman, Shane Weinmann, Emma Ferreira, Lawrence Kidd, Rasangi S. C. Suraweera, Emma J. Ferreira, Emily H. M. Budd, Gabriela J. Martin Robinson, Graeme Burt, Dhvani R. Joshipara, Bethany Roberts, William M. R. Hamilton, Abish Kunnath Kodakkat, Suhao Yap, Laurence J. W. Peters, Tomas Grundy, Megan R. Perkins, Stephanie J. Raybould, Prashant P. Verghese, Lucy Chambers, John Millwood Hargrave, Joseph D. Watson, James A. Robertson, Kiran Doddanarase Gowda, Emma J. A. Jenkins, Sophie R. Tillman, Matthew G. Williams, Isabella C. Broughton, Kathryn A. Singh, Ben J. Jones, Saskia Port, Abdallah Khalil, Luke J. Mason, Olivia Coombs, Mohamed Elbahnasy, Hannah Shereef, Samantha Black, Amr Barghout, Ishan Wijesinghe, Cafer Yuruk, Katy M. Plant, Ogbonna J. Eya, Aine McCurry, Jessica Cheng, Namratha E. Mathai, Verity Brooker, Clare Smedley, Alexander Malin, Kieran Walker, Jillian Scott, Niamh R. Davies‐Branch, Sarah F. Meredith, Aaron J. McClatchey, Rachel E. Poustie, Gareth W. Lipton, Charlie D. Johnson, Christopher A. Brennan, Kevin P. A. Dibb, Sorcha C. Heelan, Prashant Kumar, Jennifer C. Newton, Iain Mactier, Mark A. Tait, Kirsty L. McCrorie, Laura Orr, Syed Mehboob Mazhar, Sharandeep Singh, Seamus G. Crumley, Niamh Hughes, Shivani Sharda, Shane N. Campbell, Simon Chitnis, Christina L. Dunn, Emily Stratton, Jakub Foytl, Rebecca J. Brown, Samuel Bennett, Joshua Edwards, Katherine Francis, Sophie E. Horrocks, Steven A. McClune, Sam Talbot, Tina V. Bylinski, Charles A. Flanders, Sally J. Thomson, Anna te Water Naudé, Gary S. Neill, Caitlyn L. Taylor, Kirsty Morrison, Inez Armstrong, Catherine Cook, Lucie Weatherall, Paul J. C. Wilson, Mohamed Abada, Regina Graham, Rachel S. Newby, Genoveva Gomez Gomez de la Torre, Jonathan D. Pobjoy, Emily Frost, Evelyn A. E. Jones, Akbar Karimi, Rebecca Miller, Matthew South, Declan F. McKernan, Rachael B. Allen, Kerry Chrystal, Nicola Powley, Charlotte O'Driscoll, Gareth Gamble, Orlaith F. C. McManus, Lok H. A. Lin, Bruce Liu, James Wicker, David W. Tuffley, Babak M. Barzi, Christopher Blenkharn, Emma Pearson, Charles A. Wallis, Christopher Parsons, Daniel C. Hathaway, Johannes J. F. Marais, Arrenvir Jaspal‐Mander, Rhiannon Harling, Charles A. McVickers, Jessica Scott, Jonathan W. Dennis, Emily S. London, Priyanka V. Kamble, Aakar Thapa, Aaliya J. Gilbert, Annabel O. Lloyd‐Thomas, Samay Mellor, Stuart Connal, Lylah Irshad, Michael T. Lee, Chee Hwai Lim, Tamanna Shikh‐bahaei, Jack M. Williams, James Collis, Elspeth M. Cumber, Oliver Arscott, Robyn A. Lee, Jack F. Ingham, Callum R. Taylor, Lucy F. Charig, Bradley Postill, Eleanor C. O. Taylor, Stephanie Walsh, Caren Chu, Melanie Hosken, Rosie Lauste, Oliver O'Keeffe, Hans van Huellen, Sandeep Sudan, Alice R. Ball, James Wright, Keri N. Joslyn, Jimmy Siu, Fraser Cohen, Rikesh Dattani‐Patel, Tian Zhe Wong, Ching Cheng Daniel Hsieh, Katharine J. Richardson, Jan Hansel, Oscar Pope, Panagiotis Mastrogiannopoulos, Joshua T. Moore, Juraj Hajnik, Benjamin Huggon, Tarek Mouket, Sotonye Ogan, Sidra Shah, Luke P. Patterson, Benjamin Sykes, Murray R. Williams

**Affiliations:** ^1^ Borders General Hospital Melrose UK; ^2^ South East Scotland School of Anaesthesia UK; ^3^ St John's Hospital Livingston UK; ^4^ Western General Hospital Edinburgh UK; ^5^ Usher Institute, University of Edinburgh Edinburgh UK

When interpreted correctly, waveform capnography can prevent morbidity and mortality during airway management. However, misidentification of capnography as other waveforms (e.g. pressure or flow) continues to cause preventable deaths [1] and has been implicated in a Coroner's Regulation 28 report [2]. Non‐standardised monitor displays increase the risk of human error, leading to clinical delays or misjudgements [3]. Despite the capnography recommendations from the Association of Anaesthetists [4], Project for Universal Management of Airways and capnography safety campaigns, preventable deaths persist [5]. System design (removing the possibility of error) is the most effective form of error prevention [6]. In recognition of this, the Safe Anaesthesia Liaison Group (SALG) recommends standardising waveform capnography as a white solid filled‐in graph at the bottom of the monitor display [7]. It is not known how widely this recommendation has been adopted. We aimed to establish the extent of variation in waveform capnography across the UK and assess compliance with the SALG standard.

We devised a survey to collect capnography waveform and equipment data from participating hospitals (online Supporting Information Appendix S1). A website (https://cavastudy.co.uk) was established for hospital registration and respondent survey access. Participation was voluntary and open to all 420 NHS hospitals that provide anaesthesia services [1].

Survey respondents were asked to identify distinct clinical areas in their hospital where waveform capnography was used, establish the number of different waveforms in each area, and categorise them according to 11 colours, two waveform types and three screen locations. These variants were chosen from a pilot survey in south‐east Scotland. Departmental clinical directors of participating hospitals were asked to agree to their hospital's participation and state their personal awareness of the SALG standard. The survey was not anonymised. Research Ethics Committee and Caldicott Guardian approval were not required. Investigators registered the project via their local governance teams.

Survey responses were collected from 9 September 2024 to 31 October 2024 using Microsoft Forms (Microsoft, Redmond, WA, USA). Analysis was conducted in R Studio (R version 4.4.1; R Foundation, Vienna, Austria).

Data were received from 138/420 (33%) eligible hospitals (which were part of 65 NHS Trusts/health boards). We analysed 9052 individual capnography waveforms and identified 36 variants across nine colours, two morphologies (line and filled in) and three monitor locations (top, middle and bottom). The most common waveform was a white line at the bottom of the screen, followed by the SALG standard and then a white line in the middle. The remaining capnographs varied considerably in morphology (Table 1 and Fig. 1).

**Table 1:** Top five waveform capnography variants reported most frequently.

Rank	Capnography design	Waveform description	Prevalence
			n = 9052
1	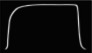	Colour: White Type: Line Location: Bottom of screen	2496 (27.6%)
2*	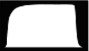	Colour: White Type: Filled In Location: Bottom of screen	1385 (15.3%)
3	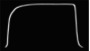	Colour: White Type: Line Location: Middle of screen	747 (8.25%)
4	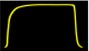	Colour: Yellow Type: Line Location: Bottom of screen	637 (7.04%)
5	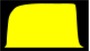	Colour: Yellow Type: Filled in Location: Top of screen	496 (5.48%)

* SALG standard.

**Figure 1:**
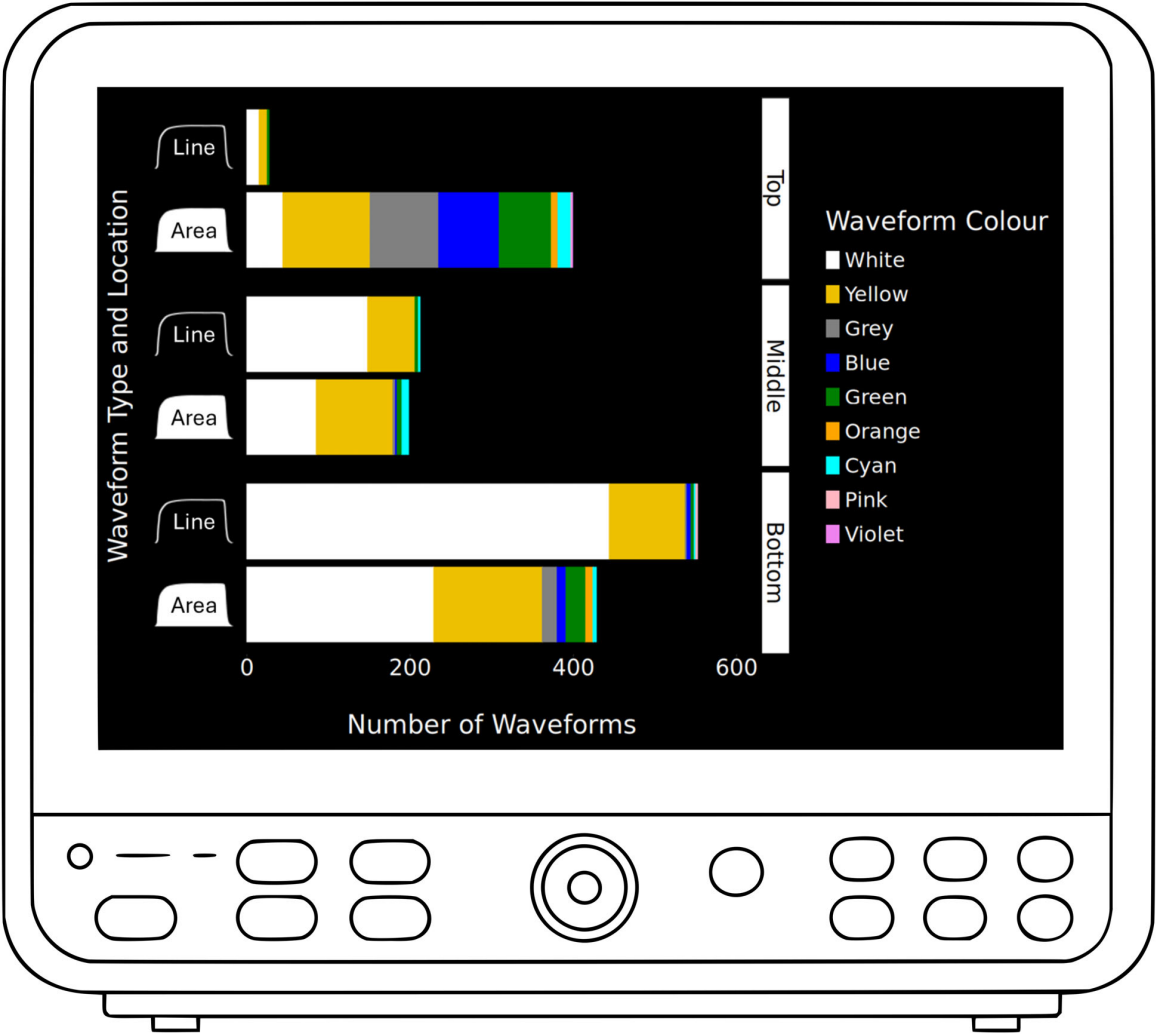
Quantities of display variants presented according to colour, trace type and location on screen. Colours were white, yellow, grey, blue, green, orange, cyan, pink and violet. Types were either a line or filled area. Locations were logged as top, middle or bottom of the screen.

In 585/1816 (32%) clinical areas, monitors displayed other waveforms (e.g. pressure or flow), with identical morphology to capnography. The median (IQR [range]) of waveform variants per hospital was 4 (2–5.25 [1–13]). Across Trusts/health boards with multiple hospital sites, variants increased to 6 (4–8 [1–15]).

Monitoring equipment service manuals were examined for customisation options to meet the SALG standard: 5008/9143 (54.5%) machines were user‐modifiable to the standard; 4054/9143 (44.5%) required manufacturer modification; and 81/9143 (1%) were unmodifiable due to hardware limitations. The SALG standard was known to 77/102 (75%) of clinical directors.

This survey highlights potential patient safety risks relating to variability in waveform capnography across UK hospitals. Both capnography waveform heterogeneity and ambiguity of non‐capnography waveforms increase the risk of misinterpretation, potentially leading to errors in situational awareness. Such misinterpretations contribute to over 80% of anaesthesia‐related adverse events, primarily due to failures in perceiving and comprehending critical information [8]. The risk is particularly high for clinicians moving between different hospital areas and rotating within Trusts/heath boards.

Despite a low response rate (33%) from eligible hospitals, 36 variants of capnograph is concerning. A higher rate of participation may have identified even greater variation. We did not assess the impact of variation on clinical performance or error rates, and we did not explore the practical or financial implications of national capnography waveform standardisation.

Despite publication of a Regulation 28 Coroner's report and the Royal College of Anaesthetists' national response, patients have continued to die due to unrecognised oesophageal intubation in the UK [5]. Although 54.5% of monitoring machines were user‐modifiable and 75% of clinical leads were aware of the SALG standard, only 15.3% of waveforms conformed to it. System‐level design limitations also contribute to the problem, with 44.5% of machines requiring manufacturer modification (online Supporting Information Table S1). This substantiates the Coroner's concern and suggests that clinicians and individual Trusts/health boards cannot be relied upon alone to implement the necessary standardisation.

Our findings provide a snapshot of the extent of waveform capnography variation in the UK. Mandated change via regulatory intervention (as was performed for gas cylinder colours) is necessary to achieve national standardisation, whilst specialist airway societies and safety groups should continue to work collaboratively with machine manufacturers to facilitate this process and strive to achieve the SALG national standard for waveform capnography.

## Supporting information


**Appendix S1.** Survey forms as text taken from Microsoft Forms.

**Appendix S2.** The CaVa UK collaborators.

**Table S1.** Device manufacturers encountered and their proportion of use.
