# Supplementary material for: A UK‐wide survey evaluation of capnography variation
Source: Anaesthesia. 2025 Mar 17;80(6):716–9. doi: 10.1111/anae.16603 (PMC12066889; doi:10.1111/anae.16603)
Supplement: Supplementary file 1 — Appendix S1. Survey forms as text taken from Microsoft Forms. [file ANAE-80-716-s001.docx]

**Appendix S1:** Survey forms as text taken from Microsoft Forms

**CaVa UK Site Enrolment Form**

This form is used to enrol each anaesthetic department to CAVA UK for further dissemination of survey materials.
Most hospitals will have a single department with unified governance. In some cases, however, a single hospital site may have multiple governance teams.

Please agree on the division of departments locally prior to enrolment to avoid duplication.

1. **Site Lead Investigator Name***Enter your answer*
2. **Site Lead Investigator Email***Enter your answer*
3. **Name of Hospital***Enter your answer*
4. **Name of Trust/Health Board***Enter your answer*
5. **Supporting Regional Trainee Research Network (If applicable)***Enter your answer*

**CaVa Survey UK Site Clinical Director Form**

Governance surrounding waveform capnography at your site.

1. **Name of Department Lead***Enter your answer*
2. **Hospital***Enter your answer*
3. **Health Board or Trust***Enter your answer*
4. **Are you/were you aware of the SALG statement on waveform capnography standardisation?**
   - Yes
   - No
5. **Do you consent to data collection in your department and for subsequent publication of any results?**
   - Yes
   - No

**CaVa UK Area Form**

Form to fill for each area in your department.

1. **Trust or Health Board**
   *Enter your answer*
2. **Hospital Site**
   *Enter your answer*
3. **Area Name**
   *Enter your answer*
4. **Clinical Area Type**
   *Select your answer*
5. *Anaesthetic rooms*
6. *Operating theatres*
7. *Recovery bays*
8. *Emergency department bays*
9. *Transfer monitors*
10. *ICU bed spaces*
11. *Dental suites*
12. *ECT suites*
13. *Radiology scanning suites*
14. *Interventional radiology suites*

**Capnography Used**
Where two variants are used at the same station, please use the ventilator display as Variant 1. Any duplication should be logged as another variant.

1. **Variant 1** (e.g. 32 RAB)
   *Enter your answer*
2. **Variant 2** (e.g. 21 CLT or N/A)
   *Enter your answer*
3. **Variant 3** (e.g. 5 GrAM or N/A)
   *Enter your answer*
4. **Other Variants** (please state numbers of machines and waveform code or NA)
   *Enter your answer*
5. **How many machines display waveforms identical in morphology to the CO_2_ waveform?** (e.g. Pressure/Volume/Agent) (none = 0)
   *Enter your answer*

**Capnography Display Equipment Log**
To better understand the hardware and software limitations across the UK, please include the names of machines used in this location and whether they can be modified to the SALG standard.

1. **Capnography Display Equipment and numbers used** (e.g. 12 Draeger Primus)
   *Enter your answer*
