# Supplementary material for: A UK‐wide survey evaluation of capnography variation
Source: Anaesthesia. 2025 Mar 17;80(6):716–9. doi: 10.1111/anae.16603 (PMC12066889; doi:10.1111/anae.16603)
Supplement: Supplementary file 2 — Appendix S2. The CaVa UK collaborators. [file ANAE-80-716-s002.docx]

Appendix S2: The CaVa UK Collaborators

### Alder Hey Children’s NHS Foundation Trust

#### Alder Hey Children’s Hospital

##### Site Co-Leads

Thomas Colville
Ashoke J Shah

**Data Collection Assistance**
Sarah Thornley

### Aneurin Bevan University LHB

#### Grange University Hospital

##### Site Lead

Ashley Davies

**Data Collection Assistance**
Sara Jones

#### Maindiff Court Hospital

##### Site Lead

Ashley Davies

#### Neville Hall Hospital

##### Site Lead

Ashley Davies

**Data Collection Assistance**
Radharetnasivan Meiarasu

#### Royal Gwent Hospital

##### Site Lead

Ashley Davies

#### St Woolos Hospital

##### Site Lead

Ashley Davies

#### Ysbyty Ystrad Fawr

##### Site Lead

Ashley Davies

### Bedfordshire Hospitals NHS Foundation Trust

#### Bedford Hospital

##### Site Lead

Ashton D Dsouza

### Belfast Health & Social Care Trust

#### Belfast City Hospital

##### Site Lead

Simon N Smith

#### Mater Hospital

##### Site Lead

Jay P Dorman

#### Royal Victoria Hospital

##### Site Co-Leads

Katy Foreman
Kirsty S McClelland
Emily F Reid

**Data Collection Assistance**
Ellen A Gorman
Charlotte Spiers

### Betsi Cadwaladr University LHB

#### Wrexham Maelor

##### Site Lead

Alison K Hare

**Data Collection Assistance**
Thomas Abberton
Aisha Abdelrahman
Georgia Ashley
Michael Gardiner
Fiona Glarvey
Syed Hussain
Alexander Poyner
Rebecca Williams

#### Ysbyty Gwynedd

##### Site Lead

Maeve Graham

### Blackpool Teaching Hospitals NHS Foundation Trust

#### Blackpool Victoria Hospital

##### Site Co-Leads

Sandor Bako
Anish Chandrasekar

**Data Collection Assistance**
Laura Scott
Amy Spicer
Dan Turner
Sean Williams

### Bolton NHS Foundation Trust

#### Royal Bolton Hospital

##### Site Co-Leads

Victoria Courtice
Kumar Saurabh
Vivek Trivedi

**Data Collection Assistance**
Tanveer A Ansari
Lucy Brown

### Cardiff & Vale University LHB

#### University Hospital Llandough

##### Site Lead

Vaishali Vyas

#### University Hospital of Wales

##### Site Lead

Vaishali Vyas

**Data Collection Assistance**
Chloe Butel
Oskar Mahony
Amy M Nixon

### Chesterfield Royal Hospital NHS Foundation Trust

#### Chesterfield Royal Hospital

##### Site Lead

Nathan P Griffiths

**Data Collection Assistance**
Anand D Padmakumar

### Countess of Chester Hospital NHS Foundation Trust

#### Countess of Chester Hospital

##### Site Co-Leads

Thomas Colville
Rhianna S Jones

### County Durham and Darlington NHS Foundation Trust

#### Darlington Memorial Hospital

##### Site Lead

Jothika Thimappa

**Data Collection Assistance**
Lucy Rothwell

#### University Hospital North Durham

##### Site Lead

Mary Leese

### Cwm Taf Morgannwg University Local Health Board

#### Princess of Wales Hospital

##### Site Lead

Harriet L White

**Data Collection Assistance**
Jessica H Phillips

#### Royal Glamorgan Hospital

##### Site Lead

Mostafa A Elsayed

### Dorset County Hospital NHS Foundation Trust

#### Dorset County Hospital

##### Site Lead

John EC Melville

### East Kent Hospitals University NHS Foundation Trust

#### Queen Elizabeth the Queen Mother Hospital

##### Site Lead

Tony Hodgetts

**Data Collection Assistance**
Angela E Munteanu

### East Lancashire Hospitals NHS Trust

#### Burnley General Hospital

##### Site Lead

Robert Brown

**Data Collection Assistance**
Aidan Butler
Angus Isham

#### Royal Blackburn Hospital

##### Site Lead

Kate Lloyd

### East Sussex Healthcare NHS Trust

#### Conquest Hospital

##### Site Lead

James G Cowman

#### Eastbourne District General Hospital

##### Site Lead

Shane Weinmann

### Gloucestershire Hospitals NHS Foundation Trust

#### Cheltenham General Hospital

##### Site Co-Leads

Emma Ferreira
Lawrence Kidd
Rasangi SC Suraweera

#### Gloucester Royal Hospital

##### Site Co-Leads

Emma J Ferreira
Lawrence Kidd
Rasangi SC Suraweera

### Great Western Hospitals NHS Foundation Trust

#### Great Western Hospital

##### Site Lead

Emily HM Budd

**Data Collection Assistance**
Gabriela J Martin Robinson
Graeme Burt

### Hampshire Hospitals NHS Foundation Trust

#### Royal Hampshire County Hospital

##### Site Lead

Dhvani R Joshipara

**Data Collection Assistance**
Bethany Roberts

### Hywel Dda University LHB

#### Glangwilli Hospital

##### Site Co-Leads

William MR Hamilton
Abish Kunnath Kodakkat

#### Prince Philip Hospital

##### Site Lead

Abish Kunnath Kodakkat

### Kettering General Hospital NHS Foundation Trust

#### Kettering General Hospital

##### Site Lead

Suhao Yap

**Data Collection Assistance**
Laurence JW Peters

### Lancashire Teaching Hospitals NHS Foundation Trust

#### Chorley Hospital

##### Site Lead

Tomas Grundy

**Data Collection Assistance**
Megan R Perkins

#### Preston Hospital

##### Site Lead

Tomas Grundy

### Liverpool Heart and Chest Hospital NHS Foundation Trust

#### Liverpool Heart and Chest Hospital

##### Site Co-Leads

Thomas Colville
Stephanie J Raybould

**Data Collection Assistance**
Prashant P Verghese

### Liverpool University Hospitals NHS Foundation Trust

#### Aintree University Hospital

##### Site Co-Leads

Lucy Chambers
Thomas Colville

**Data Collection Assistance**
John Millwood Hargrave
Joseph D Watson

### Liverpool Women’s NHS Foundation Trust

#### Liverpool Women’s Hospital

##### Site Co-Leads

Thomas Colville
James A Robertson

**Data Collection Assistance**
Kiran Doddanarase Gowda

### London North West University Healthcare NHS Trust

#### Central Middlesex Hospital

##### Site Lead

Emma JA Jenkins

#### Ealing Hospital

##### Site Lead

Sophie R Tillman

#### Northwick Park Hospital

##### Site Lead

Matthew G Williams

**Data Collection Assistance**
Isabella C Broughton
Kathryn A Singh

### Manchester University NHS Foundation Trust

#### Manchester Royal Infirmary

##### Site Co-Leads

Ben J Jones
Saskia Port

#### North Manchester General Hospital

##### Site Co-Leads

Abdallah Khalil
Luke J Mason

#### Oxford Road

##### Site Lead

Olivia Coombs

#### Royal Eye Hospital

##### Site Co-Leads

Ben J Jones
Saskia Port

#### St Mary’s Hospital

##### Site Lead

Olivia Coombs

#### Trafford General Hospital

##### Site Co-Leads

Ben J Jones
Saskia Port

#### Wythenshawe Hospital

##### Site Co-Leads

Mohamed Elbahnasy
Hannah Shereef

### Medway NHS Foundation Trust

#### Medway Maritime Hospital

##### Site Lead

Samantha Black

**Data Collection Assistance**
Amr Barghout
Ishan Wijesinghe
Cafer Yuruk

### Mersey and West Lancashire Teaching Hospitals NHS Trust

#### Ormskirk General Hospital

##### Site Co-Leads

Thomas Colville
Katy M Plant

**Data Collection Assistance**
Ogbonna J Eya
Aine McCurry

#### Southport General Hospital

##### Site Co-Leads

Thomas Colville
Katy M Plant

**Data Collection Assistance**
Jessica Cheng
Ogbonna J Eya
Aine McCurry

#### St Helen’s Hospital

##### Site Co-Leads

Thomas Colville
Namratha E Mathai

#### Whiston Hospital

##### Site Co-Leads

Thomas Colville
Namratha E Mathai

**Data Collection Assistance**
Verity Brooker

### Mid Cheshire Hospitals NHS Foundation Trust

#### Leighton Hospital

##### Site Co-Leads

Thomas Colville
Clare Smedley

**Data Collection Assistance**
Alexander Malin
Kieran Walker

### NHS Ayrshire and Arran

#### University Hospital Crosshouse

##### Site Lead

Jillian Scott

**Data Collection Assistance**
Niamh R Davies-Branch
Sarah F Meredith

### NHS Fife

#### Queen Margaret Hospital

##### Site Lead

Aaron J McClatchey

**Data Collection Assistance**
Rachel E Poustie

#### Victoria Hospital Kirkcaldy

##### Site Lead

Aaron J McClatchey

### NHS Golden Jubilee

#### Golden Jubilee National Hospital

##### Site Lead

Gareth W Lipton

### NHS Grampian

#### Aberdeen Maternity Hospital

##### Site Lead

Charlie D Johnson

**Data Collection Assistance**
Christopher A Brennan

#### Aberdeen Royal Infirmary

##### Site Lead

Charlie D Johnson

**Data Collection Assistance**
Kevin PA Dibb
Sorcha C Heelan

#### Royal Aberdeen Children’s Hospital

##### Site Lead

Charlie D Johnson

**Data Collection Assistance**
Sorcha C Heelan

#### Royal Cornhill Hospital

##### Site Lead

Charlie D Johnson

#### Woodend Hospital

##### Site Lead

Charlie D Johnson

### NHS Greater Glasgow and Clyde

#### Gartnaval Hospital

##### Site Lead

Prashant Kumar

**Data Collection Assistance**
Jennifer C Newton

#### Glasgow Royal Infirmary

##### Site Co-Leads

Iain Mactier
Mark A Tait

**Data Collection Assistance**
Kirsty L McCrorie

#### Institute of Neurological Sciences

##### Site Co-Leads

Laura Orr
Mark A Tait

**Data Collection Assistance**
Syed Mehboob Mazhar

#### Inverclyde Royal Hospital

##### Site Lead

Sharandeep Singh

#### New Victoria Hospital

##### Site Lead

Prashant Kumar

**Data Collection Assistance**
Seamus G Crumley

#### Queen Elizabeth University Hospital

##### Site Lead

Prashant Kumar

**Data Collection Assistance**
Niamh Hughes
Shivani Sharda

#### Royal Hospital for Children

##### Site Lead

Shane N Campbell

**Data Collection Assistance**
Simon Chitnis
Christina L Dunn

#### Stobhill Hospital

##### Site Lead

Iain Mactier

### NHS Highland

#### National Treatment Centre

##### Site Lead

Emily Stratton

#### New Craigs Hospital

##### Site Lead

Emily Stratton

#### Raigmore Hospital

##### Site Lead

Emily Stratton

### NHS Lothian

#### Royal Edinburgh Hospital

##### Site Lead

Jakub Foytl

#### Royal Hospital for Children and Young People

##### Site Lead

Jakub Foytl

**Data Collection Assistance**
Rebecca J Brown

#### Royal Infirmary of Edinburgh

##### Site Lead

Jakub Foytl

**Data Collection Assistance**
Samuel Bennett
Rebecca J Brown
Joshua Edwards
Katherine Francis
Sophie E Horrocks
Steven A McClune

#### St John’s Hospital

##### Site Lead

Sam Talbot

**Data Collection Assistance**
Tina V Bylinski
Charles A Flanders
Sally J Thomson

#### Western General Hospital

##### Site Lead

Anna te Water Naudé

**Data Collection Assistance**
Gary S Neill

### NHS Shetland

#### Gilbert Bain Hospital

##### Site Lead

Caitlyn L Taylor

### NHS Tayside

#### Ninewells Hospital

##### Site Lead

Kirsty Morrison

### North Bristol NHS Trust

#### Southmead Hospital

##### Site Lead

Inez Armstrong

**Data Collection Assistance**
Catherine Cook

### Northampton General Hospital NHS Trust

#### Northampton General Hospital

##### Site Lead

Lucie Weatherall

### Northern Care Alliance NHS Foundation Trust

#### Royal Oldham Hospital

##### Site Lead

Paul JC Wilson

### Nottingham University Hospitals NHS Trust

#### Nottingham City Hospital

##### Site Co-Leads

Mohamed Abada
Regina Graham

#### Queens Medical Centre

##### Site Lead

Rachel S Newby

### Salisbury NHS Foundation Trust

#### Salisbury District Hospital

##### Site Lead

Genoveva Gomez Gomez de la Torre

### Sherwood Forest Hospitals NHS Foundation Trust

#### Kings Mill Hospital

##### Site Lead

Jonathan D Pobjoy

**Data Collection Assistance**
Emily Frost
Evelyn AE Jones
Akbar Karimi
Rebecca Miller
Matthew South

### South Eastern Health & Social Care Trust

#### Ulster Hospital Dundonald

##### Site Lead

Declan F McKernan

**Data Collection Assistance**
Rachael B Allen
Kerry Chrystal

### South Tees Hospitals NHS Foundation Trust

#### Friarage Hospital

##### Site Lead

Nicola Powley

#### James Cook Hospital

##### Site Lead

Nicola Powley

**Data Collection Assistance**
Charlotte O’Driscoll

### Southern Health & Social Care Trust

#### Craigavon Area Hospital

##### Site Lead

Gareth Gamble

**Data Collection Assistance**
Orlaith FC McManus

### Stockport NHS Foundation Trust

#### Stepping Hill Hospital

##### Site Co-Leads

Lok HA Lin
Bruce Liu

### Surrey and Sussex Healthcare NHS Trust

#### Crawley Hospital

##### Site Lead

James Wicker

**Data Collection Assistance**
David W Tuffley

#### East Surrey Hospital

##### Site Lead

James Wicker

**Data Collection Assistance**
Babak M Barzi
Christopher Blenkharn
Emma Pearson
David W Tuffley
Charles A Wallis

### Swansea Bay University Health Board

#### Emergency Medical Retrieval and Transfer Service Wales

##### Site Lead

Christopher Parsons

#### Morriston Hospital

##### Site Co-Leads

Daniel C Hathaway
Johannes JF Marais

**Data Collection Assistance**
Arrenvir Jaspal-Mander

#### Neath Port Talbot Hospital

##### Site Co-Leads

Rhiannon Harling
Daniel C Hathaway

#### Singleton Hospital

##### Site Co-Leads

Rhiannon Harling
Daniel C Hathaway

### The Newcastle Upon Tyne Hospitals NHS Foundation Trust

#### Freeman Hospital

##### Site Lead

Charles A McVickers

#### Royal Victoria Infirmary

##### Site Lead

Jessica Scott

**Data Collection Assistance**
Jonathan W Dennis

### The Walton Centre NHS Foundation Trust

#### The Walton Centre

##### Site Co-Leads

Thomas Colville
Emily S London

### United Lincolnshire Hospitals NHS Trust

#### Grantham and District Hospital

##### Site Lead

Priyanka V Kamble

#### Pilgrim Hospital

##### Site Lead

Aakar Thapa

### University College London Hospitals NHS Foundation Trust

#### Grafton Way Building

##### Site Lead

Aaliya J Gilbert

**Data Collection Assistance**
Annabel O Lloyd-Thomas
Samay Mellor

#### National Hospital for Neurology & Neurosurgery

##### Site Lead

Stuart Connal

#### University College Hospital

##### Site Lead

Aaliya J Gilbert

**Data Collection Assistance**
Lylah Irshad
Michael T Lee
Chee Hwai Lim
Annabel O Lloyd-Thomas
Samay Mellor
Tamanna Shikh-bahaei

#### Westmoreland Street

##### Site Lead

Aaliya J Gilbert

**Data Collection Assistance**
Jack M Williams

### University Hospital Southampton NHS Foundation Trust

#### Princess Anne Hospital

##### Site Co-Leads

James Collis
Elspeth M Cumber

**Data Collection Assistance**
Oliver Arscott

#### University Hospital Southampton

##### Site Co-Leads

James Collis
Elspeth M Cumber

**Data Collection Assistance**
Oliver Arscott
Robyn A Lee

### University Hospitals Bristol and Weston NHS Foundation Trust

#### Bristol Eye Hospital

##### Site Co-Leads

Jack F Ingham
Callum R Taylor

#### Bristol Royal Infirmary

##### Site Co-Leads

Jack F Ingham
Callum R Taylor

#### Saint Michael’s Hospital

##### Site Co-Leads

Jack F Ingham
Callum R Taylor

#### South Bristol Community Hospital

##### Site Co-Leads

Jack F Ingham
Callum R Taylor

#### Weston General Hospital

##### Site Co-Leads

Jack F Ingham
Callum R Taylor

### University Hospitals Dorset NHS Foundation Trust

#### Poole Hospital

##### Site Lead

Lucy F Charig

**Data Collection Assistance**
Bradley Postill
Eleanor CO Taylor
Stephanie Walsh

#### Royal Bournemouth Hospital

##### Site Lead

Lucy F Charig

**Data Collection Assistance**
Caren Chu
Melanie Hosken
Rosie Lauste
Oliver O’Keeffe

### University Hospitals Sussex NHS Foundation Trust

#### Lewes Victoria Hospital

##### Site Lead

Hans van Huellen

**Data Collection Assistance**
Sandeep Sudan

#### Princess Royal Hospital

##### Site Lead

Hans van Huellen

#### Royal Alexandra Children’s Hospital

##### Site Lead

Hans van Huellen

**Data Collection Assistance**
Alice R Ball
James Wright

#### Royal Sussex County Hospital

##### Site Lead

Hans van Huellen

**Data Collection Assistance**
Alice R Ball
Keri N Joslyn
Jimmy Siu
James Wright

#### Sussex Orthopaedic Treatment Centre

##### Site Lead

Hans van Huellen

#### Worthing Hospital

##### Site Lead

Fraser Cohen

### University Hospitals of Leicester NHS Trust

#### Leicester Royal Infirmary

##### Site Co-Leads

Rikesh Dattani-Patel
Tian Zhe Wong

**Data Collection Assistance**
Ching Cheng Daniel Hsieh
Katharine J Richardson

### University Hospitals of Morecambe Bay NHS Foundation Trust

#### Royal Lancaster Infirmary

##### Site Lead

Jan Hansel

**Data Collection Assistance**
Oscar Pope

### Warrington and Halton Teaching Hospitals NHS Foundation Trust

#### Captain Sir Tom Moore Hospital

##### Site Co-Leads

Thomas Colville
Panagiotis Mastrogiannopoulos

#### Warrington Hospital

##### Site Co-Leads

Thomas Colville
Panagiotis Mastrogiannopoulos

**Data Collection Assistance**
Joshua T Moore

### Wirral University Teaching Hospital NHS Foundation Trust

#### Arrowe Park Hospital

##### Site Co-Leads

Thomas Colville
Juraj Hajnik

#### Clatterbridge hospital

##### Site Co-Leads

Thomas Colville
Juraj Hajnik

#### Wirral Women and Children’s Hospital

##### Site Co-Leads

Thomas Colville
Juraj Hajnik

### York and Scarborough Teaching Hospitals NHS Foundation Trust

#### Bridlington Hospital

##### Site Lead

Benjamin Huggon

#### Scarborough General Hospital

##### Site Lead

Tarek Mouket

**Data Collection Assistance**
Sotonye Ogan
Sidra Shah

#### York Hospital

##### Site Co-Leads

Luke P Patterson
Benjamin Sykes

**Data Collection Assistance**
Murray R Williams
