# Supplementary material for: A UK‐wide survey evaluation of capnography variation
Source: Anaesthesia. 2025 Mar 17;80(6):716–9. doi: 10.1111/anae.16603 (PMC12066889; doi:10.1111/anae.16603)
Supplement: Supplementary file 3 — Table S1. Device manufacturers encountered and their proportion of use. [file ANAE-80-716-s003.docx]

**Table S1:** Device manufacturers encountered and their proportion of use. Values are number (proportion)

| **Manufacturer** | **n = 9179** |
| --- | --- |
| Philips | 2940 (32%) |
| Draeger | 2554 (27.8%) |
| GE | 1818 (19.8%) |
| Mindray | 1216 (13.3%) |
| Maquet | 176 (1.9%) |
| Fukuda | 171 (1.9%) |
| Spacelabs | 152 (1.7%) |
| Proact | 59 (0.64%) |
| Hamilton | 28 (0.31%) |
| Newtech | 12 (0.13%) |
| Masimo | 10 (0.11%) |
| Iradimed | 8 (0.09%) |
| Medtronic | 8 (0.09%) |
| Zoll | 7 (0.08%) |
| Penlon | 6 (0.07%) |
| MIPM | 3 (0.03%) |
| Edan | 2 (0.02%) |
| HP | 2 (0.02%) |
| Prolite | 2 (0.02%) |
| Medrad | 1 (0.01%) |
